# A Suggestive Case

**Published:** 1889-08

**Authors:** 


### A Suggestive Case.

Mrs. C, aged about fifty, has been wearing a full set of teeth on rubber plate about twenty years. They were worn all this time till within the last two and a half years; during this time there has been considerable soreness at the posterior border of the upper plate; this was relieved by cutting away the border of the plate about two lines and rounding it off at the upper edge. There was also a little irritation of the mucous membrane at the buccal border of the same plate which was also relieved by dressing. The irritation at both these points was undoubtedly due to the settling, though very slow, of the alveolar ridge.

A greater and apparently more serious difficulty was found on the tongue on the left side and about opposite the first and second molars on the lower denture. This consisted of an ulcer of about a third of an inch in diameter, and a line in depth. Round about this there was considerable induration. It had been in this condition for more than two years, and all this time undergoing treatment by a physician, or physicians rather, for several had treated it. The statement was made that it was probably malignant and would necessitate the removal of a part, if not all of the tongue. The patient had been for a considerable time in great trouble about it and was much depressed.

She was questioned, as to whether the tongue was ever caught between the teeth, to which she replied that she was not aware that there was any thing of the kind. Upon examination it was found that the molars of both the upper and lower dentures of the left side had been ground quite smooth on the masticating surfaces and very squarely, so that the inner edges came very accurately together, quite to the lingual surfaces of these teeth, and it was found that the tongue was, in masticating, constantly though imperceptibly, caught in this close-fitting bite; thus inducing and continuing the ulcer on the tongue. The angles of these were now rounded off and dressed so that the tongue was not caught. Immediate improvement of the affected part resulted and in a short time was entirely well.

Two points are brought to notice by this case: the first is that care should be taken by dentists in the construction of artificial dentures, whether full or partial sets, to avoid all points of irritation to the soft parts either by roughness of any part, or by unequal pressure, especially at the edges of the plates, or as in the case above described, by biting and bruising the soft parts in the occlusion of the teeth; and secondly more care should be exercised by both dentists and physicians, and especially the latter, in respect to mouth affections, and especially those connected with the teeth, as to diagnosis and prognosis. The case above given was treated by a physician for about two years under the impression that it was rapidly tending to malignancy, and he was of the opinion that it had already attained that condition, and that nothing short of the removal of a part, if not the whole of the tongue would prevent a fatal result. In this case two or three physicians had the same opinion. Two dentists examined it, and were quite befogged as to the cause or nature of the affection. Had there been in this case a favorable condition for the development of cancer or malignant tumors, such disease might have taken place, but that it had not yet assumed that form was manifest by the results; and still further, the more obvious indications of malignancy were not present.

It is well that due caution be always exercised in the examination and pronouncing on affections arising from diseased teeth, for they are often very obscure, treacherous, and persistant. Error has been too often committed in prematurely announcing malignancy, but on the other hand of denying its presence when strong evidence was present to that effect. Malignant diseases should be studied and better understood by all who in a medical or surgical aspect may have any thing to do with them.

